# *Staphylococcus aureus* but not *Listeria monocytogenes* adapt to triclosan and adaptation correlates with increased *fabI* expression and *agr* deficiency

**DOI:** 10.1186/1471-2180-13-177

**Published:** 2013-07-30

**Authors:** Lene Nørby Nielsen, Marianne Halberg Larsen, Sissel Skovgaard, Vicky Kastbjerg, Henrik Westh, Lone Gram, Hanne Ingmer

**Affiliations:** 1 Department of Veterinary Disease Biology, Faculty of Health and Medical Sciences, University of Copenhagen, Copenhagen, Denmark; 2 Hvidovre Hospital, Department of Clinical Microbiology, Copenhagen, Denmark; 3 Department of Clinical Medicin, Faculty of Health and Medical Sciences, University of Copenhagen, Copenhagen, Denmark; 4 National Food Institute, Technical University of Denmark, Copenhagen, Denmark

**Keywords:** *Staphylocooccus aureus*, *Listeria monocytogenes*, Adaptation, Triclosan

## Abstract

**Background:**

The ability of pathogens to adapt to the widely used biocide, triclosan, varies substantially. The purpose of the study was to examine bacterial adaptation over an extended period of time to low increments of triclosan concentrations. Focus was two human pathogens, *S*. *aureus* and *L*. *monocytogenes* that previously have displayed inherent high and low adaptability, respectively.

**Results:**

Three strains of *L*. *monocytogenes* and two strains of *S*. *aureus* including the community-acquired USA300 were exposed to increasing, sub-lethal concentrations of triclosan in triclosan-containing agar gradients. Following 25 days of exposure on agar plates to sub-lethal concentrations of triclosan with a twofold concentration increase every second day, minimum inhibitory concentration (MIC) for *S*. *aureus* increased from 0.125 (8325–4) and 0.0625 (USA 300) mg/L to 4 mg/L. The MIC of all three *L*. *monocytogenes* strains was initially 4 mg/L and remained unaltered by the exposure. The adapted *S*. *aureus* isolates retained normal colony size but displayed increased expression of *fabI* encoding an essential enzyme in bacterial fatty acid synthesis. Also, they displayed decreased or no expression of the virulence associated *agrC* of the *agr* quorum sensing system. While most adapted strains of USA300 carried mutations in *fabI*, none of the adapted strains of 8325–4 did.

**Conclusions:**

Adaptability to triclosan varies substantially between Gram positive human pathogens. *S*. *aureus* displayed an intrinsically lower MIC for triclosan compared to *L*. *monocytogenes* but was easily adapted leading to the same MIC as *L*. *monocytogenes*. Even though all adapted *S*. *aureus* strains over-expressed *fabI* and eliminated expression of the *agr* quorum sensing system, adaptation in USA300 involved *fabI* mutations whereas this was not the case for 8325–4. Thus, adaptation to triclosan by *S*. *aureus* appears to involve multiple genetic pathways.

## Background

Biocides are used extensively in our society for cleaning and disinfection purposes. Triclosan is a bisphenol biocide and has been widely used for the past 30 years, for a broad range of applications. Triclosan is used in many household products such as soaps, shampoos, detergents, tooth paste and it is also used to prevent or reduce bacterial contamination in many cosmetics [1,2]. These products typically contain concentrations of triclosan ranging from 0.1 to 0.3% by weight, corresponding to 1000 to 3000 mg/L [1].

In the food industry, triclosan has been incorporated into different types of plastics including food storage containers and chopping boards [3]. In these food associated bacteria including pathogens such as *Listeria monocytogenes* may be repeatedly exposed to the compound [4]. In the hospital environment pathogens may encounter triclosan when it is employed in surgical scrubs, sutures and soaps [5,6]. For patients that are carriers of methicillin resistant *Staphylococcus aureus* (MRSA), triclosan body wash has been used to eradicate MRSA carriage prior to surgery [5,7].

The extensive use of triclosan is also reflected in its environmental presence. Several studies have detected triclosan in the external environment such as in streams and rivers [8,9], surface waters downstream of wastewater treatment plants [8], biosolids [10] and accumulating in higher organisms such as snails and fish [1,11]. Concentrations in the natural aquatic environment are detected to a maxima of 0.0023 mg/L and freshwater sediments range from 0.8 – 53 mg/kg [12]. As the concentration of triclosan increases in the environment, one may fear that bacteria with decreased susceptibility to the compound will be selected for [13,14].

In contrast to many biocides, triclosan has a specific cellular target, namely the enoyl-acyl carrier protein reductase isoform, FabI, an essential enzyme in bacterial fatty acid synthesis [15]. At low concentrations, triclosan inhibits fatty acid synthesis by inhibiting FabI that uses NADH to reduce a double bond during each cycle of bacterial fatty acid elongation [16]. At higher concentration, the bisphenol causes gross damage to bacterial cell membranes and disrupts protein and lipid biosynthesis [17,18].

While resistance to biocides at working concentrations is rare, increased tolerance to triclosan is commonly observed for many bacterial species including *S*. *aureus*[16,19-23]. There are multiple pathways by which bacterial cells may become more tolerant to triclosan including point mutations in *fabI*[15,24,25], overexpression of FabI [25,26] or increased efflux pump activity extruding the compound [27]. Interestingly, the latter activity has been associated with quinolone resistance in *E*. *coli*, *Pseudomonas aeruginosa* and *S*. *aureus*[15,28-30]. Other phenotypic changes accompanying triclosan tolerance have been noted. In *S*. *aureus* exposure to triclosan has given rise to small colony variants (SCV) that in addition to altered metabolism and virulence gene expression are resistant to gentamicin [31]. Interestingly, short term exposure of *Listeria monocytogenes* to triclosan in sub-lethal concentrations did not result in an altered MIC towards triclosan but caused cells to develop resistance to gentamicin and other aminoglycosides [32]. This finding suggests that an adaptation to triclosan occured in these bacteria even though the triclosan MIC was not affected.

Since the literature suggests that Gram positive bacteria have different adaptation mechanisms to triclosan it is important to address this question in a controlled study using the same adaption methodology and using small increments in triclosan concentrations over an extended period of time to mimic the repeated exposure in e.g. hospital or food processing environments.

## Methods

### Bacteria and growth conditions

The following strains were used in the adaptation studies: *S*. *aureus* 8325–4 [33]*S*. *aureus* USA300 LAC [34], *L*. *monocytogenes* F2365 (LMG 23356) [35], *L*. *monocytogenes* N53-1 [36] and *L*. *monocytogenes* EGD (obtained from Werner Goebel, Biozentrum, University of Würzburg, Germany). For the hemolysin assay *S*. *aureus* RN4220 (26) was used. In the assesment of efflux activity, strains JCM 16555 (*qacA*) and JCM 16556 (*qacB*) were included as controls (both kindly provided by Japan Collection of Microorganisms, RIKEN BRC which is participating in the National BioResource Project of the MEXT, Japan). *S*. *aureus* strains were grown in Mueller Hinton (MH) broth (Oxoid, Denmark) or Tryptic Soy broth (TSB) (Oxoid) or on corresponding agar plates (MHA, TSA) (Oxoid). *L*. *monocytogenes* was grown in Brain Heart Infusion (BHI) broth (Oxoid) or on BHI agar plates (Oxoid). All cultures were incubated under aerobic conditions at 37°C and broth cultures were shaken at 200 rpm. Triclosan (Irgasan; Sigma-Aldrich) was dissolved (1000 mg/L) in 96% ethanol (Kemotyl, Denmark) for adaptation on plates and in 70% ethanol for the adaptation in broth, and further diluted in growth medium and added to the samples when indicated.

### Adaptation on gradient agar plates

A Triclosan-gradient was constructed in TSA (*S*. *aureus*) or BHI-agar (*L*. *monocytogenes*) in four-sided petri dishes. Each bacterial isolate was inoculated as streaks on these plates (7 replicas per strain) and the plates incubated 1 day at 37°C. After incubation, the colony mass was swabbed from 1 cm at the leading edge of growth (at the highest triclosan concentration) and re-streaked on agar plates with the same triclosan gradient. If there was bacterial growth on the entire inoculation streak the bacteria were streaked on agar plates were the triclosan concentration in the bottom layer was twice as high. If no growth was observed the bacteria were streaked again on agar plates with the same triclosan gradient. The initial concentration of triclosan in the bottom layer was 0.002 for *S*. *aureus* 8325–4 and all *L*. *monocytogenes* strains, and 0.0002 μg/ml for *S*. *aureus* USA300.

For controls, the five bacterial strains were sub-cultivated on agar plates without triclosan as often as bacteria grown on triclosan gradient plates. When it was no longer possible to increase the concentration of triclosan in the gradient plates the 7 replicas and 2 controls were frozen at −80°C as single colonies.

After adaptation all *S*. *aureus* isolates were confirmed as *S*. *aureus* by use of Rapid Staph Test kit (Oxoid) and all *L*. *monocytogenes* isolates confirmed on Rapid’L. mono plates (Bio-Rad).

### Adaptation in broth

*Listeria monocytogenes* EGD and N53-1 were grown sequentially with triclosan in BHI as described previously [37] with minor modifications. Following five transfers in BHI without triclosan, each of five individually passaged sets of both strains were exposed to 0.05 μg/ml triclosan and two to 0.7% ethanol as negative control. The experiment was conducted for approx. 70 transfers with 1:100 fold dilutions, giving a total of 420–490 bacterial generations. All selection lines were streaked daily on BHI-agar to check purity and occasionally verified as *Listeria* by cultivation on Listeria selective agar base (Oxoid) supplemented with modified *Listeria* selective supplement (Oxoid).

### Estimation of triclosan tolerance, MIC and minimum bacteriocidal concentration (MBC)

The MIC of triclosan was determined following the recommendations of the British Society of Antimicrobial Chemotherapy using broth microdilutions (CLSI). A stock solution of triclosan was prepared in advance and a doubling dilution range from 0.0156 - 8 mg/l triclosan in Muller-Hinton bouillon (MHB) was made for each experiment. Triclosan dilution (100 μl) and 100 μl of an over night bacterial suspension adjusted to 10^6 CFU/ml was mixed in each well. The MIC was determined as the lowest concentration that inhibited visible growth after 24 hours. Bacterial growth in the highest dilution of 96% ethanol used was also tested and did not differ visually from growth in MHB alone. From each well (0.0625 mg/l and above) 10 μl was, after 24 hours incubation, spotted on to Muller-Hinton agar (MHA) plates containing no triclosan. The MBC was read as the lowest concentration with no growth after 48 hours. All MIC determinations were made with two biological and two technical replicates and MIC is given as the mean.

### Sequencing of *fabI*

Genomic DNA of *S*. *aureus* was isolated using the boiling method as described previously [38] and *fabI* amplified by PCR (with a mixture of 25 μl DreamTaq™Green PCR Master Mix ([2×]), 1 μM of each primer, 1 μl DNA template and water to a total volume of 50 μl). Primers adapted from those designed for the amplification of the staphylococcal *fabI* gene and putative promoter region were used in 50 μl PCRs (fab1F: tgttccgcatggagatacac and fab1R: taaggactaattctgtggatgt) [39]. The PCR was performed with an initial 5 min denaturation at 95°C, followed by 35 cycles of denaturation at 94°C for 30 s, annealing at 50°C for 30 s and extension at 72°C for 45 s, followed by a final extension step at 72°C for 5 min. The PCR products were purified using 10 units Exonuclease I (ExoI) and 1 unit FastAP™Thermosensitive Alkaline Phosphatase mixed with 5 μl PCR product, then incubated at 37°C for 15 min and 85°C for 15 min. The purified PCR products were sequenced at both strands using Macrogenservice.

### RNA extraction and northern hybridization

Cells of *S*. *aureus* were grown to mid logarithmic growth phase (OD600 = 0.6-0.8) in MH broth and samples were immediately cooled in ice-water bath. The bacterial cells were lysed using Fast Prep FP120 instrument (BIO101, ThermoSavent) for 45 s at speed 6.0. Total RNA was extracted from the cells using RNeasy mini kit (Qiagen, Denmark) according to the manufacturer’s directions. Analysis of transcripts was made as previously described [40]. Hybridization probes were generated by PCR from chromosomal DNA of *S*. *aureus* 8325–4 using specific primers for the *fabI* gene (fab2F: atgttaaatcttgaaaacaaaac and fab2R: ttatttaattgcgtggaatccgc) [39], the *mepA* gene (mepAF: ggaaacttcgcgattgca and mepAR: gcacaaagtgactcagca), the *norA* gene (norAF: tgtttgcagttggccaca and norAR: cgccacccgtaatagcaa) and *rnaIII* (*rnaIII*F: ggggatcacagagatgtgatg and *rnaIII*R: gggcatagcactgagtccaagg) (TAG Copenhagen A/S, Denmark). RNA extracted from at least two independent experiments was analysed.

### Hemolytic activity

Hemolytic activity was evaluated on sheep blood as described previously [41].

### Assessment of efflux activity

Efflux pump activity was analysed by ethidium bromide (EtBr) assay as described previously [42]. Isolates were grown for 24 h at 37°C on TSA containing EtBr (1 mg/L), followed by inspection under UV light.

### Deoxyribonuclease (DNAse) activity

DNAse activity was determined semi-quantitatively on DNase agar (Oxoid). After overnight incubation, clearing zones after addition of 1 M hydrochloric acid were measured using calipers.

### Metabolic activity using XTT

(2,3-bis-[2-methoxy-4-nitro-5-sulfopheny l]-2H-tetrazolium-5-carboxanilide) (Sigma). A colorimetric assay where the colorless XTT is reduced to the water soluble formazan dye by metabolically active cells was used to compare the metabolic activity between triclosan adapted strains and non adapted strains. Cultures were grown over night, harvested by centrifugation (8000 rpm, 10 min) (Eppendorf minispin (F-45-12-11) and resuspended in XTT. The XTT suspension was incubated at 37°C, dark and with shake for 3 h. The cultures were centrifuged (8000 rpm, 10 min) (Eppendorf minispin (F-45-12-11) and OD_490_ measured on supernatant.

### Attachment

*S*. *aureus* strains were grown in TSB to OD_600_ 0.05 and 100 μl pre-culture was added to a 96-well microtiter plate (Sarstedt) and incubated overnight at 37°C. Then, each well was washed three times with 200 μl physiological saline and stained with 0.1% crystal violet for 30 min. The crystal violet was washed out and each well washed three times with water. The amount of attachment was determined after dissolving the crystal violet with 96% ethanol for 30 min and read by spectrophotometer at 490 nm.

### Antimicrobial susceptibility

MICs of gentamicin and vancomycin were measured using Etest (bioMerieux-Diagnostics, Denmark).

## Results and discussion

### *S*. *aureus* but not *L*. *monocytogenes* adapts to triclosan

To compare the adaptability of *L*. *monocytogenes* and *S*. *aureus* to triclosan, both species were exposed to daily sub-cultivations on agar plates without or with gradient concentrations of triclosan (see Methods). For *S*. *aureus* also parallel passage of 7 single colonies of each of the two strains USA300 and 8325–4 were made. *S*. *aureus* 8325–4 is a common laboratory strain while USA300 is a community associated MRSA that is causing serious infections in the USA and many other countries [43]. After 25 days of daily passages the highest concentration of triclosan in the top agar layer that supported growth of *S*. *aureus* was 4 mg/L triclosan for both 8325–4 and for USA300. Colony material from each of the 7 exposed cultures and 2 controls (eg. sub-cultivated without triclosan) was stored for further characterization. The MIC and MBC were determined for the isolates. Interestingly, both strains of *S*. *aureus* increased their MIC about 16 fold and also the MBC was substantially increased compared to the frozen stock and strains passaged without triclosan (Table 1). The adapted strains were subsequently passaged in the absence of triclosan and the MIC of the adapted cells remained unaltered indicating that genetic changes were responsible for the altered MIC (data not shown).

**Table 1:** **Minimum inhibitory concentration** (**MIC**) **and minimum bacteriocidal concentration** (**MBC**) **values for *S***. ***aureus S***. ***aureus *USA300**, ***L***. ***monocytogenes *EGD**, ***L***. ***monocytogenes *N53**-**1 and *L***. ***monocytogenes *F2365**

**Strain**	**MIC 24 h (mg/L)**	**MIC 48 h (mg/L)**	**MBC 24 h (mg/L)**	**MBC 48 h (mg/L)**
***S*. *aureus *8325-4**				
Freezer stock	0.125	0.25	2	2
Passaged 1	0.125	0.25	2	2
Passaged 2	0.125	0.25	2	2
Adapted 1	2	4	8	8
Adapted 2	2	4	8	8
Adapted 3	1	2	8	8
Adapted 4	2	4	8	8
Adapted 5	2	4	8	8
Adapted 6	2	4	8	8
Adapted 7	2	2	8	8
***S*. *aureus *USA300**				
Freezer stock	0.0625	0.125	1	2
Passaged 1	0.125	0.25	4	4
Passaged 2	0.125	0.25	4	4
Adapted 1	2	2	8	8
Adapted 2	2	2	8	8
Adapted 3	2	4	8	8
Adapted 4	2	4	8	8
Adapted 5	2	4	8	8
Adapted 6	2	4	8	8
Adapted 7	2	4	8	8
***L*. *monocytogenes *EGD**				
Freezer stock	2	4	8	8
Passaged 1	2	4	8	8
Passaged 2	2	4	8	8
Adapted 1	4	4	8	8
Adapted 2	4	4	8	8
Adapted 3	4	4	8	8
Adapted 4	4	4	8	8
Adapted 5	4	4	8	8
Adapted 6	4	4	8	8
Adapted 7	4	4	8	8
***L*. *monocytogenes *N53-1**				
Freezer stock	2	4	8	8
Passaged 1	2	4	8	8
Passaged 2	2	4	8	8
Adapted 1	4	4	8	8
Adapted 2	4	4	8	8
Adapted 3	4	4	8	8
Adapted 4	4	4	8	8
Adapted 5	4	4	8	8
Adapted 6	4	4	8	8
Adapted 7	4	4	8	8
***L*. *monocytogenes *F2365**				
Freezer stock	2	4	8	8
Passaged 1	2	4	8	8
Passaged 2	2	4	8	8
Adapted 1	4	4	8	8
Adapted 2	4	4	8	8
Adapted 3	4	4	8	8
Adapted 4	4	4	8	8
Adapted 5	4	4	8	8
Adapted 6	4	4	8	8
Adapted 7	4	4	8	8

When *L*. *monocytogenes* strains EGD, F2365 (LMG23356) and N53-1 were passaged similarly, the highest concentration of triclosan in the top agar layer supporting growth was 1.28 mg/L for N53-1 and EGD whereas F2365 was able to grow in the presence of 4 mg/L triclosan in the top layer. *L*. *monocytogenes* EGD is a laboratory strain whereas N53-1 was isolated from fish processing industry and F2365 (LMG23356) is from a Mexican-style soft cheese [35,36]. The adapted isolates of *L*. *monocytogenes* F2365 and EGD multiplied only at slightly higher triclosan concentration than the controls (freeze stock and strains passaged with no triclosan) (Table 1). In order to address if adaptation of *L*. *monocytogenes* may occur during growth in liquid broth *L*. *monocytogenes* EGD and N53-1 were passaged with increasing concentrations of triclosan. However, after ten transfers in 4.8 mg/L, none of the ten selection lines were able to grow in 6.4 mg/L (data not shown). Again it was found that the MIC of the exposed strains was similar to the triclosan unexposed bacteria (data not shown). Thus, the inherent tolerance of two Gram positive bacterial species towards triclosan differed substantially with *L*. *monocytogenes* being 16 fold more tolerant than *S*. *aureus*. In contrast, *S*. *aureus* readily adapts to triclosan and the final MIC of the adapted *S*. *aureus* cells approximates that of the unadapted *L*. *monocytogenes*.

### Efflux pump activity is not altered in triclosan adapted *S*. *aureus* strains

**Figure 1:**
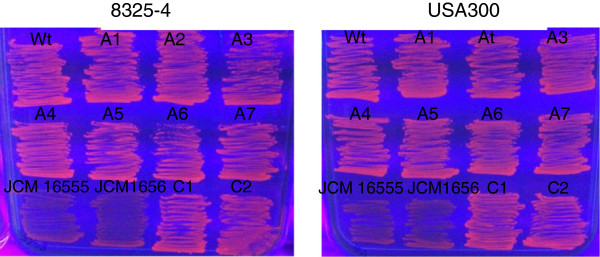
**EtBr efflux activity in triclosan exposed *S*. *aureus *cells.** Isolates were grown for 24 h at 37°C on TSA containing EtBr (1 μg/ml), followed by inspection for fluorescence under UV light as described previously. Wt (freeze stock), C1 and C2 (two controls passed on TSA without triclosan), A1-A7 (7 triclosan adapted strains). Strains JCM 16555 (*qacA*) and JCM 16556 (*qacB*) (lower left), that overexpress efflux pumps, were included as controls.

With the aim of determining if the adaptation processes are the same in the two strains of *S*. *aureus*, namely the laboratory strain 8325–4 and the clinical isolate USA300, the adapted isolates were characterized. Previously, enhanced expression of the AcrAB efflux pump has been associated with increased tolerance to triclosan in *E*. *coli*[27]. To address if efflux pump activity may be part of the adaptation process in *S*. *aureus* strains, the efflux activity was examined by monitoring extrusion of ethidium bromide [44]. As shown in Figure 1 noobvious differences in ethidium bromide staining of triclosan tolerant and adapted strains were observed indicating that they are equally able to extrude the compound. Transcription of *norA* and *mepA* were also measured and no differences between triclosan adapted strains and their controls were observed (data not shown).

### *fabI* sequence and expression

Increased tolerance to triclosan may be a consequence of mutations in *fabI* encoding the enoyl-ACP reductase that is essential for *S*. *aureus* fatty acid biosynthesis [25]. To address if the gene was mutated in the adapted *S*. *aureus* strains, the *fabI* of both triclosan adapted and unadapted cells of *S*. *aureus* 8325–4 and USA300 was sequenced. For the triclosan adapted isolates of strain 8325–4 no mutations were found in the 1358 bp DNA region covering the *fabI* gene and promoter region when compared to the sequence of the freezer stock of 8425–4 and the passaged control cells. In contrast, several mutations occurred in the *fabI* gene and promoter region of the adapted USA300 isolates compared to the passaged but un-exposed control cells and the published sequence of USA300 (Table 2). *fabI* mutations previously associated with triclosan tolerance are a substitution of a phenylalanine for a cysteine or serine at position 204 (F204C) (F204S) [25,45], a glycine for a serine at position 23 (G23S) [16] or an aspartic acid for a threonine in position 101 [45]. The F204C mutation does not allow triclosan to form a stable complex between NAD + and FabI-triclosan contributing to the decrease in sensitivity [25]. Mutations in position 204 in four of the seven triclosan adapted USA300 isolates were also identified here. Two isolates had the previously identified F204C substitution but in addition substitutions in F204L and F204S were also found (Table 2). Further a substitution in A24T was identified in one isolate, D101Y in another isolate and I193F in again two other isolates. No amino acid changes were observed in any of the isolates passaged without triclosan. It is clear from this and previous studies [25,45] that a mutation in position 204 is important for *S*. *aureus* in adaptation to triclosan. However, the mutation does not seem to be essential as other mutations were also discovered and even strains with no mutations in *fabI* were adapted to triclosan. Analysis of recognition, interaction and binding of triclosan to *S*. *aureus* FabI showed that F204 is one of the interacting residues located within the substrate-binding loop of FabI [46]. This could be an explanation for the frequently identified mutations in this position that probably prevent optimal binding and activity of triclosan to FabI.

**Table 2:** **Aminoacid substitutions in FabI of USA300 strains passaged either in the absence** (**C1 and C2**) **or presence** (**A1 through A7**) **of triclosan compared to the genome sequence** (**34**)

**Aminoacid number (from gene start)**	**C1**	**C2**	**A1**	**A2**	**A3**	**A4**	**A5**	**A6**	**A7**
**Upstream of *fabI***									
−74								M → R	
−32							I → T		
−19									V → D
***fabI***									
24			A → T						
101								D → Y	
193				I → F	I → F				
204			F → L			F → S	F → C	F → C	

Minus positions are upstream of the protein start. *Abbreviations* for amino acids are: *M* Methionine, *R* Arginine, *I* Isoleucine, *T* Threonine, *V* Valine, *D* aspartic acid, *A* alanine, *N* asparagine, *F* Phenyl alanine, *C* cysteine, *L* Leucine, *S* Serine.

**Figure 2:**
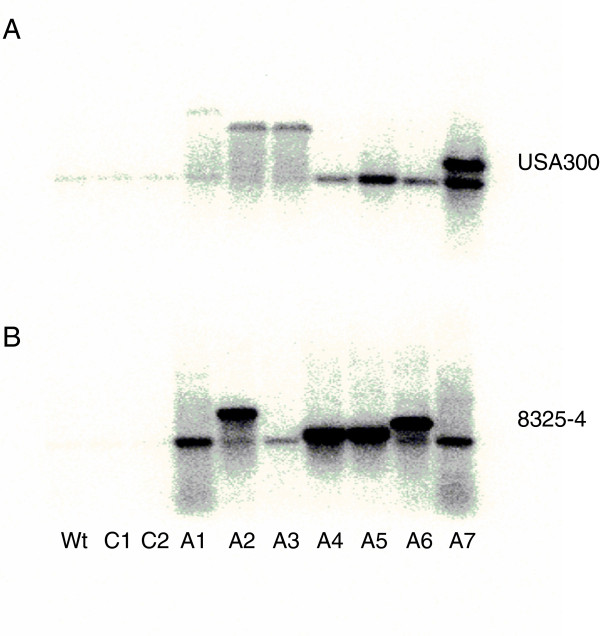
***fabI *expression in triclosan exposed *S*. *aureus *cells.** Northern blot showing *fabI* expression with **A**. USA300 and **B**. 8325-4. From left: wt (freeze stock), C1 and C2 (two controls passed on TSA without triclosan), A1-A7 (7 triclosan adapted strains).

Increased expression of the wild type FabI has also been related to enhanced tolerance to triclosan in *S*. *aureus*[39] although to a lesser degree than mutations in the *fabI* gene [25]. To examine if altered expression of *fabI* is related to triclosan adaptation particularly in strain 8325–4, where no *fabI* mutations were detected, the amounts of *fabI* mRNA produced during growth in MH medium were compared (Figure 2). In all the adapted isolates of strains 8325–4, *fabI* was strongly overexpressed indicating that this may contribute to the decreased triclosan susceptibility. Also, in most of the adapted isolates of strain USA300 increased *fabI* expression was seen. This indicates that in these strains both mutations in and expression of *fabI* may influence triclosan susceptibility. Interestingly, it was also observed that in the un-adapted strains, *fabI* was expressed as a monocistronic transcript but in several of the adapted strains larger transcripts appeared. The *fabI* gene is located 94 bp downstream of an open reading frame (designated USA300HOU_0968) oriented in the same direction as *fabI*. Sequencing of USA300HOU_0968 and intergenic region in the adapted isolates of USA300 revealed various mutations but none of them appeared in more than one isolate (Table 2). However, it is predicted that USA300HOU_0968 and *fabI* form an operon and that mutations in USA300HOU_0968 and *fabI* enhance expression of the operon.

### Virulence factor expression is altered in triclosan adapted strains

Previous studies have indicated that exposure of *S*. *aureus* to triclosan may lead to a SCV phenotype characterized by slow growth and pin point small colonies [31,47]. The common underlying factor in induction of the SCV phenotype is reduced bacterial energy generation/transport leading to reduced cell wall synthesis and virulence factor production including hemolysis, coagulase and DNAse. Importantly, both the adapted strain 8325–4 and USA300 formed colonies of regular size when compared to unexposed cells (data not shown). In order to further confirm that respiration in the adapted strains was comparable to wild type cells, enzymatic activity using XTT assay was monitored and no differences were found between adapted, passaged and control strains (freeze stocks) (data not shown). In contrast to previous studies, it was not found that triclosan exposure lead to SCV. One explanation may be that this study used TSA growth medium compared to the less nutritious MHA used in other studies [31,47]. Thus, *S*. *aureus* adapts to triclosan and interestingly different strains use different measures to decrease susceptibility.

**Figure 3:**
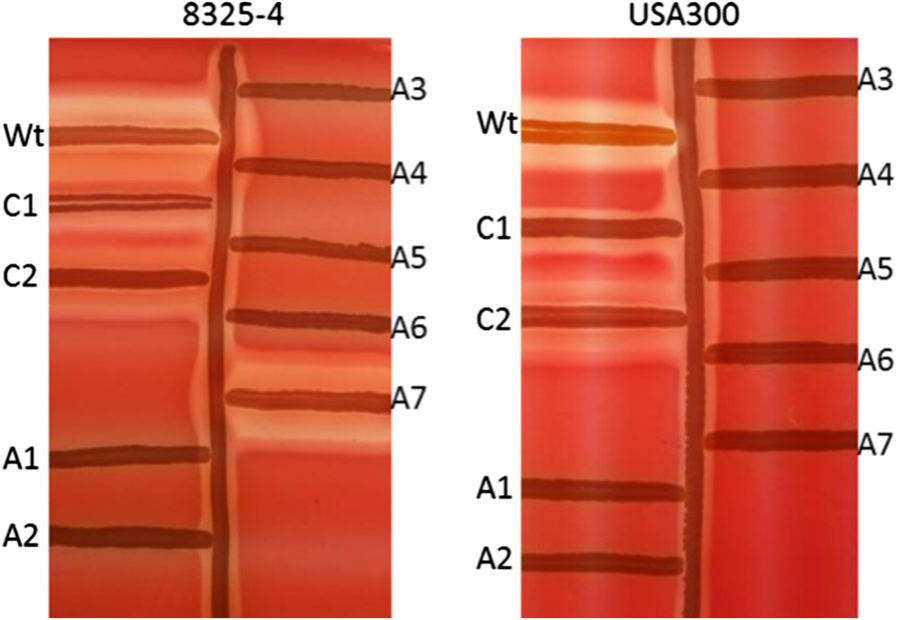
**Hemolytic activity of *S*. *aureus *on sheep blood.** RN4220, a strong beta-hemolysin producer, is streaked vertically, and at right wt (freeze stock), C1 and C2 (two controls passed on TSA without triclosan). At right from top and down A1-A7 (7 triclosan adapted strains). Beta-hemolysin forms a tubid zone of hemolysis surrounding the vertical streak of RN4220. Delta-hemolysis and beta-hemolysis are synergistic, producing a clear hemolysis where they intersect.

**Figure 4:**
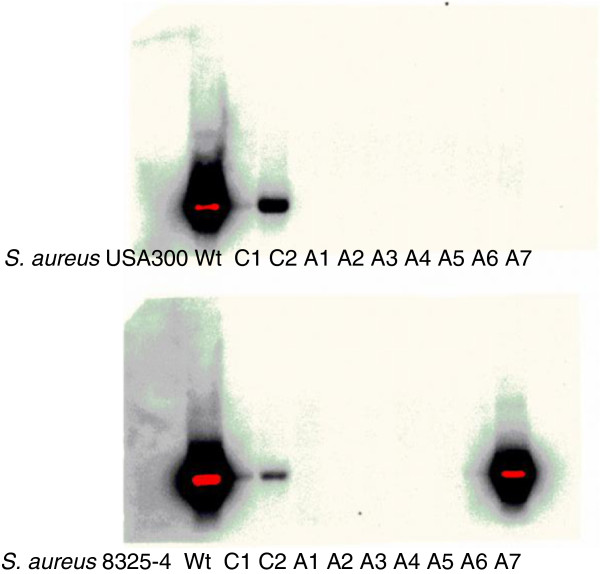
**RNAIII expression in triclosan exposed *S*. *aureus *cells.** Northern blot displaying *rnaIII* expression from USA300 and 8325-4. Starting from left: wt (freeze stock), C1 and C2 (two controls passed on TSA without triclosan), A1-A7 (7 triclosan adapted strains).

Although no SCVs were observed in response to triclosan in this study the effect of adaptation on virulence gene expression was examined [48]. One of the key virulence factors in *S*. *aureus* is α-hemolysin encoded by *hla*. Hemolysin activity is conveniently evaluated on sheep blood agar plates and results showed that all but one (8325–4 A7) of the adapted strains of *S*. *aureus* USA 300 and 8325–4 displayed little or no hemolytic activity in contrast to the freezer stocks of the strains. Curiously, the isolates that had been passaged in the absence of triclosan also expressed less hemolytic activity compared to the original stock but greater activity than the triclosan adapted isolates (Figure 3). *hla* is regulated by the effector molecule RNAIII and where *hla* is decreased a reduction in RNAIII level is also expected. The level of RNAIII expression was therefore examined by Northern blot analysis and was found to be almost abolished in the adapted strains compared to passaged and wild type stock strains (Figure 4). Only one adapted strain (8325–4 A7) showed RNAIII expression correlating well with the hemolysis observed for the same strain (Figure 3). In addition to the toxins, *S*. *aureus* produces an array of degradation enzymes including nucleases. Using a semi-quantitative agar-based assay DNAse activity was monitored but no differences between the adapted isolates and the controls (passaged and wt strains) were observed (data not shown). This contrasts a recent study where triclosan exposure resulted in SCVs expressing reduced DNase activity [49]. In agreement with previous findings, a significant reduction in attachment to plastic surfaces was observed for the adapted strains of both *S*. *aureus* 8325–4 and USA300 compared to the control strains (data not shown) [49].

### Antibiotic resistance

Sub-lethal exposure of triclosan has been shown to decrease susceptibility to gentamicin and other aminoglycosides in *L*. *monocytogenes*[32]. It was examined if that also was the case for *S*. *aureus* strains passaged in the presence of triclosan. Here, no alterations were observed for (gentamicin (0.5 μg/ml for non-adapted and adapted *S*. *aureus* 8325–3 and 1 μg/ml for non-adapted and adapted *S*. *aureus* USA300 strains) and the glycopeptide antibiotic vancomycin (2 μg/ml for *S*. *aureus* 8325–4 *and S*. *aureus* USA300 with no differences between non-adapted and adapted)). These findings agree with an earlier study where a triclosan tolerant *S*. *aureus* was examined for its susceptibility to a number of antibiotics belonging to different classes but with no changes compared to the triclosan sensitive strain [50].

## Conclusion

In conclusion, this study shows that *S*. *aureus* has intrinsically lower MIC to triclosan as compared to *L*. *monocytogenes* but can be adapted to the same tolerance level. The adaptation in *S*. *aureus* is not accompanied by antibiotic resistance but rather reduced expression of the central virulence regulatory RNA, RNAIII and in most cases overexpression of *fabI* encoding the enoyl-acyl carrier protein reductase isoform, the target of triclosan. However, the phenotypic diversity of triclosan adapted *S*. *aureus* strains suggests that multiple adaptation pathways may lead to decreased triclosan susceptibility. This notion was confirmed by sequencing of the *fabI* gene and promoter region revealing that while most adapted strains of USA300 carried mutations in this region none of the 8325–4 strains did. Thus, multiple mutational events may adapt *S*. *aureus* to triclosan and the genetic path selected in adaptation may be strain specific.

## Competing interests

The authors declare that they have no competing interests.

## Authors’ contributions

LNN participated in the study design, carried out laboratory work, analysed the data, and drafted the manuscript. MHL conceived the study, analysed the data and edited the manuscript. SS participated in the study design, productive discussions and edited the manuscript. VK participated in the study design, carried out laboratory work, analysed the data and edited the manuscript. HW and LG participated in the study design and edited the manuscript. HI conceived the study, edited the manuscript and received funding for the research. All authors have read and approved the final manuscript.
